# Vaccination Coverages Among Splenectomized Patients: A Retrospective Study from an Italian Southern Province

**DOI:** 10.3390/vaccines13020138

**Published:** 2025-01-28

**Authors:** Giuseppe Di Martino, Riccardo Mazzocca, Tania Masci, Lisa Berghella, Jacopo Del Papa, Francesco D’Aloisio, Mario Messinese, Fabrizio Cedrone, Patrizia Marani Toro, Graziella Soldato

**Affiliations:** 1 Unit of Epidemiology and Health Statistics, Local Health Authority of Pescara, 65100 Pescara, Italy; 2 Department of Medicine and Ageing Sciences, “G. d’Annunzio” University of Chieti-Pescara, 66100 Chieti, Italy; 3 Unit of Hygiene, Epidemiology and Public Health, Local Health Authority of Pescara, 65100 Pescara, Italy; riccardo.mazzocca@asl.pe.it (R.M.); tania.masci@asl.pe.it (T.M.); lisa.berghella@asl.pe.it (L.B.); jacopo.delpapa@asl.pe.it (J.D.P.); francesco.daloisio@asl.pe.it (F.D.); mario.messinese@asl.pe.it (M.M.); patrizia.maranitoro@asl.pe.it (P.M.T.); graziella.soldato@asl.pe.it (G.S.); 4 Hospital Management, Local Health Authority of Pescara, 65100 Pescara, Italy; cedronefab@gmail.com

**Keywords:** vaccine, splenectomy, public health, preventive medicine, Italy

## Abstract

Background: Splenectomized patients have a higher risk compared to the general population of developing post-splenectomy infection, particularly by capsulated bacteria. Splenectomized patients need to be vaccinated against pneumococcal diseases, meningococcal disease, and heamophilus influenzae (Hib) in order to avoid invasive bacterial diseases. This study evaluated vaccination coverages among splenectomized patients in a Southern Italian province. Methods: A retrospective study was conducted evaluating all splenectomized patients from the Pescara province from 2015 to 2023. Vaccination coverages were calculated before and after splenectomy for the following vaccines: pneumococcal disease, meningococcal disease, Hib, and COVID-19. Results: A total of 97 patients were considered during the study period. Vaccination coverages were low before surgery, but they increased after splenectomy. Higher coverages were found against pneumococcal diseases (77.3%), meninigococcal disease (58.8%), and COVID-19 (91.8%). Conclusions: Vaccination coverages among splenectomized patients in the Pescara province are not satisfying. It is imperative to implement educational measures for patients and physicians to increase vaccination coverages.

## 1. Introduction

The spleen is a fundamental organ of the human body due to its important function. It filters the blood, removes damaged and old blood cells from circulation, and regulates the amount of white and red blood cells and platelets [1]. The spleen is also important for the development of immune responses against several kinds of pathogens through both the innate and adaptative immune systems, particularly against capsulated bacteria [2]. In particular, specialized macrophages of the spleen are able to phagocytose encapsulated pathogens that are normally opsonized by antibodies. Asplenia causes the absence of these cells [3]. Some subjects are born without a spleen (congenital asplenia), while others might require its removal due to disease or damage (splenectomized patients) [3]. The most common cause of asplenia is surgical splenectomy after polytrauma, particularly after a car accident or a sports injury [1]. However, several diseases can led to hypofunction of the spleen (hyposplenism), such as hematological and immunological disorders, malaria, and infiltrative diseases (for example: sarcoidosis, amyloidosis, and cancers) [2]. Asplenia and hyposplenism are crucial risk factors for invasive bacterial infections, in particular with encapsulated bacteria such as Streptococcus, Haemophilus influenzae type b, and Neisseria meningitidis. Splenectomized/asplenic patients have a 10–50-fold higher risk compared to the general population of developing an invasive bacterial infection [4]. An invasive infection that affects splenectomized patients is also known as overwhelming post-splenectomy infection (OPSI) [4,5]. OPSI can progress from a mild flu-like illness to fulminant sepsis in a short time period, and it reports a high mortality rate (up to 50% despite intensive treatments) [6]. The infection risk rises among patients with hematological diseases, such as lymphoma and leukemia [7]. However, the majority of infections occur within the first two years after a splenectomy [8]. To reduce post-splenectomy infections, patients should be aware of the dangers of such infections, and it is mandatory to address them with preventive measures. Vaccination against Streptococcus pneumoniae, Neisseria meningitidis, and Haemophilus Influenzae type B (Hib) can help in preventing OPSI [2]. Ideally, all vaccinations should be performed on patients planning to be splenectomized at least 4–6 weeks before surgery; for patients undergoing emergency surgery, recommended vaccinations should be given within the first 72 h after surgery and no later than 2 weeks after [9]. Regarding meningococcal diseases in Italy, the following vaccinations are available: a tetravalent conjugate meningococcal vaccine against serogroups A, C, W, and Y, and vaccines against Meningococcus type B (both four-component meningococcus B vaccine, 4CMenB, and meningococcus B Factor H-binding protein vaccine, MenB-FHbp). In particular, in Italy, the MenACWY vaccine was freely proposed to all citizens from the age of 1 year. MenB was introduced in Italy from 2015, and it was actively proposed to all newborns from the year 2017 free of charge. Regarding pneumococcal diseases, the global burden of disease attributable to pneumococcus has strongly decreased thanks to the introduction of multi-valent pneumococcal vaccinations worldwide. Nonetheless, differences exist in the effectiveness of the two main types of anti-pneumococcal vaccines, 23-valent polysaccharidic (PPSV23) and conjugate (PCV), in preventing both infection and nasopharyngeal carriage [10]. The pneumococcal conjugate vaccine, initially approved for infant use in 2000, has demonstrated efficacy in preventing both IPD and non-invasive forms. From the year 2010, a 13-valent PCV was introduced in Italy with an active offer to all newborns and to high-risk patients. From 2022, a 20-valent conjugated pneumococcal vaccine (PCV20) was available, which has substituted the 13-valent conjugated vaccine. Prior to PCV20 introduction, a sequential schedule was adopted in the majority of international guidelines for preventing pneumococcal invasive diseases among high-risk patients. In particular, the sequential schedule provides a first administration of PCV, followed by a single dose of PPSV23 after at least two months. Regarding Hib, a monovalent vaccine is available. In addition, a Hib vaccine is also included in a hexavalent vaccination that is recommended to all newborns. Also, regarding COVID-19, a specific immunization schedule was recommended for splenectomized patients in compliance with international vaccination guidelines [11]. In particular, it is recommended that people receive all recommended COVID-19 vaccine doses (two doses + one booster dose). Vaccination is especially important for patients at the highest risk of severe COVID-19, including people aged 65 years and older; and people with underlying medical conditions, including immune-compromised patients and patients living in long-term care facilities. Also, pregnant people are recommended to protect themselves and their infants. So, splenectomized patients belong to immune-compromised subjects.

The aim of this study was to evaluate vaccine coverages among splenectomized patients in a province of Southern Italy. In particular, full vaccination against pneumococcal disease, meningococcal disease, and Hib disease was considered. Secondly, partial vaccination and COVID-19 vaccination were also considered. The study was conducted in this particular province that includes one of the larger hospitals of the region, and it can be an interesting point of view for the organization of vaccination in an Italian setting. In addition, no previous data on this topic from this Southern Italian region were previously published.

## 2. Materials and Methods

This was a retrospective observational cohort study that included all individuals residing or domiciled in the province of Pescara, Southern Italy. This Italian province accounts for about 320,000 inhabitants [12]. Healthcare services in this province were managed by the Local Health Authority (LHA) of Pescara that cares for all residents of the province of Pescara. This LHA was organized in three hospitals with a hub and spoke model: a tertiary referral hospital (hub) in the city of Pescara and two spoke hospitals. The hub was one of the larger hospitals of the entire Abruzzo region, and it is a referral trauma center. The two spokes are no more than 50 km away from the hub, and they are directly linked to manage critical patients and to carry out specialist procedures. The study included all patients who underwent a splenectomy in one of the three hospitals of the province. Data were collected from the LHA registry of hospital discharge records (HDRs). The HDRs included a large variety of information about patients’ demographic characteristics and hospitalization, such as gender, age, and other information such as admission and discharge date and the discharge modality. The HDRs also include the main cause of hospitalization and up to five comorbidities or complications that occurred during the admission. In addition, a maximum of six procedures or interventions performed during the hospitalization were also included. Diagnoses and procedures were coded according to the *International Classification of Disease, 9th Revision, Clinical Modification* (ICD-9-CM). Finally, the HDR also includes a diagnosis-related group (DRG) code used to classify the admission, and also reports the reimbursement cost of the admission. The study included all patients that had a hospital admission for a splenectomy, selected by ICD 41.5 code (total splenectomy) from 1 January 2015 to 31 December 2023. Only subjects living in the province of Pescara were included. The vaccination status of each patient was assessed via the Regional Vaccination Registry (RVR). The RVR is a digital vaccination registry containing information on the vaccination history of all inhabitants of the Abruzzo region. It also registers vaccinations performed in all settings, such as at a hospital, by a general practitioner, and ambulatory vaccination. The RVR record for each patient includes the date of vaccine administration, type of vaccination, trade name of the vaccine, site of injection (only for injection vaccinations), and an anamnestic questionnaire containing information on patient health status. The RVR was accessible to all vaccination ambulatories of the Abruzzo region, and its use was mandatory accordingly to Abruzzo region recommendations. For the aim of this study, the two datasets (HDR and RVR) were matched via a unique patient identifier and anonymized prior to analysis by the regional statistical office. Only patients that survived for at least thirty days after the splenectomy procedure were included in the analysis. The vaccination status of each patient was evaluated accordingly to CDC guidelines [9]. In particular, this study considered the following vaccinations:

- Two doses of Meningococcal B (MenB) vaccine;
- Two doses of Meningococcal ACWY (MenACWY) vaccine;
- One dose of Haemophilus Influenzae type B (Hib) vaccine for patients aged over 1 years of age or three does for patients that had the vaccination during the first year of life;
- Two doses of pneumococcal (PC) vaccine. In particular, one dose of 13-valent conjugate vaccine (PCV13) and one dose of 23-valent polysaccharidic vaccine (PCV23). For patients vaccinated during the year 2023, the PC vaccination was considered completed after a single dose of 20-valent conjugated vaccine (PCV20). In order to consider patients with a single dose of PCV20 as fully vaccinated, we considered the most recent guidelines as previously stated;
- In addition, despite the lack of specific indications, vaccination coverage against COVID-19 was also evaluated.

### 2.1. Statistical Analysis

Continuous variables were shown as the mean and standard deviation (SD), and categorical variables were showed as the absolute number and percentage with a relative 95% confidence interval (95%CI). Length of stay (LOS) was expressed as a median and interquartile range (IQR) accordingly to non-normal distribution of the variable. The normal distribution of continuous variables was tested with a Shapiro–Wilk’s test.

To analyze factors associated with each vaccination studied, a logistic regression model was performed for each type of vaccination, and adjusted for age, gender, and trauma. Results of logistic regression analyses were reported as an adjusted odd ratio with a relative 95% confidence interval. In addition, in order to also evaluate factors associated with partial vaccination, a multinomial logistic regression was also performed: the dependent variable was vaccination status (not vaccinated, partially vaccinated, and fully vaccinated), and results were expressed as relative risk ratios (RRRs) of each outcome with a relative 95%CI. All covariates for all statistical multivariate models were tested for multicollinearity. For all tests, a two-sided *p*-value less than 0.05 was considered statistically significant.

### 2.2. Ethical Issues

The study was conducted strictly following regulations on data management of the Regional Health Authority of the Abruzzo region and with the Italian law on privacy (Art. 20–21 DL 196/2003) published in the Official Gazette n. 190 on 14 August 2004. Data were encrypted prior to the study at the regional statistical office, where each patient was assigned a unique identification code. This code removes the possibility of tracing the patient’s identity. According to Italian law, the use of administrative data does not require any written informed consent by patients.

## 3. Results

**Figure 1:**
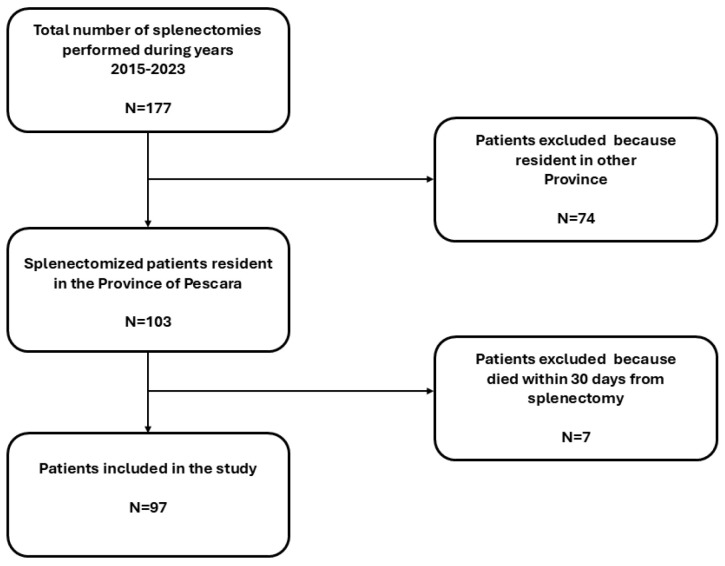
Flowchart of included patients.

Since the year 2015, a total of 177 patients underwent a total splenectomy in the province of Pescara in the three hospitals of the LHA. Among them, only 103 patients were included because they were residents in the abovementioned province. Other patients (*n* = 74) were resident in another Italian province, so they were excluded due to the lack of vaccination status information. Seven patients did not survive over 30 days after surgery and were also excluded. The flowchart of included patients is reported in Figure 1.

Among included patients, 60 were male (62.5%), and the mean age was 52.9 years (SD ± 18.9). A total of 61 splenectomies were performed as an emergency (63.5%) and 35 (36.5%) were planned. The majority of urgent splenectomies were due to traumatic circumstances (38, 62.3%). The main reason for surgery was a solid tumor (45 cases, 46.4%) followed by trauma. Most patients were discharged home (81 patients, 84.4%). Patients’ characteristics are reported in Table 1. No information on the incidence of invasive bacterial infection among included patients was available at the time of the study. The median LOS was 11 (9–17).

Regarding vaccination coverages, before splenectomy, the most frequent vaccine administered was MenACWY, with 21 patients (21.6%) immunized with two doses and 3 other patients with one dose. Similar results were achieved for Hib vaccination (13 patients, 13.4%). Only five patients (5.2%) were immunized against MenB and PCV. After splenectomy, vaccination coverages improved for all considered vaccines. In particular, the number of patients fully immunized against MenACWY was 57 (58.8%), against MenB was 57 (58.8%), against PCV was 75 (77.3%), and against Hib was 52 (53.6%). In addition, 45 patients (46.4%), 45 patients (46.4%), and 32 patients (33.0%) were partially immunized against MenACWY, MenB, and PCV, respectively. The total number of patients that received at least three doses of COVID-19 vaccination was 89 (91.8%), and seven (7.2%) patients received one or two doses. Vaccination coverages are reported in Table 2.

Considering vaccination coverages by class of age, the two patients aged less than 18 years were fully vaccinated against MenACWY, MenB, PCV, and Hib as reported in Table 3. Patients aged between 18 and 65 years reported a higher vaccination coverage for MenACWY, MenB, and PCV compared to the elderly. In particular, they reported a coverage of 64.2% for both meningitis vaccinations, 81.1% for PCV, and 32.1% for Hib. On the other hand, the elderly had a higher vaccination rate for Hib (78.6%) and COVID-19 (100%).

Regarding factors associated with becoming fully covered against a specific disease, age was significantly associated with COVID-19 vaccination (aOR = 1.10; 95%CI 1.01–1.29), and being admitted for trauma was significantly associated with Hib vaccination (aOR = 1.05; 95%CI 1.00–1.13). Results of the logistic regression analyses are reported in Table 4.

A multinomial logistic regression analysis reported similar results, with no significant factors associated with partial or full vaccination status. Results of the multinomial logistic regression analysis are reported as Supplementary Material (Table S1).

## 4. Discussion

This paper reported vaccine coverages among splenectomized patients from a Southern Italian province during the years 2015–2023. Despite no data from this region being previously published, the reported vaccination coverages were higher than highlighted in a meta-analysis published before the pandemic period [4]. In particular, a systematic review from Bianchi et al. reported 55.1% (95%CI = 41.0–69.2%) for PCV, 48.3% (95%CI = 34.3–52.3%) for Hib, 33.7% (95%CI = 23.6–43.9%) for MenACYW, and 13.3% (95%CI = 7.0–19.5%) for MenB. These results are also better than reported in the Apulia region (another Southern Italian region) [13] that reported lower coverages for all considered vaccinations. This study was a retrospective cohort study with similar data sources, performed with the same methodology. Comparing results with other European countries, including a study from Norway [3] reporting data from 2008 to 2020, showed lower coverages. This study used the same methodology (merging data on hospital admission and vaccination registry), also considering data from an infectious diseases surveillance system. Norwegian results showed low coverages for all considered diseases, with all coverages below 20%. However, these results can only be partially compared due to a different introduction period of each vaccination across countries. Also, data on COVID-19 vaccination among splenectomized patients from the Apulia region were recently published [14]: The Apulia region showed lower coverages (87.6% for the primary cycle and 61.7% for the third dose), but the study period ended in November 2022, so this difference with the present study can lead to this difference in coverages. At the same time, the methodology of the analysis was similar. However, in other countries outside Italy, vaccination coverages among splenectomized patients appear higher. As reported in a retrospective study from the UK among one hundred adults who underwent splenectomy, after 5 years of follow-up, the coverage rate of meningococcal, pneumococcal, and Hib vaccination was over 90% [15]. The strength of this study was also a comparison with a control cohort, who also had an antibody level evaluation. Similar results were highlighted from another study from the UK showing coverage against pneumococcal, meningococcal, and Hib diseases to be 91%, 80%, and 79%, respectively [16], showing the strong impact of an informative campaign conducted among general practitioners. High vaccination rates were also reported in a study conducted in Holland, with a coverage of 91% and 83%, respectively, for pneumococci and Hib [16]. This study was a prospective cohort study conducted from 2005 to 2011 and included patients evaluated in a travel medicine service. However, the main limitation of this study was the poor sample compared to the present study. In a retrospective study from Sweden that included 78 patients who underwent a splenectomy, the respective vaccination rates against pneumococcal, meningococcal, and Hib diseases were 81%, 23%, and 52% [17].

Evaluating differences in age classes, the present study showed that the elderly class reported higher coverages for Hib and COVID-19, and the middle-age class reported higher coverages for meningitis vaccination and PCV. Probably, this can be justified by the presence of meningitis vaccination in the Italian national immunization plan for adolescent and infants. Vaccination against meningitis was not proposed in the elderly. On the other hand, the elderly in Italy were vaccinated against COVID-19 before those who were younger [18]. In addition, in Italy, PCV vaccination was actively proposed to patients aged over 65 years, leading to a high coverage compared with other high-income countries. With a decree in 2017 of the Italian law on mandatory vaccination, all school-aged subjects until the age of 17 years have to be vaccinated against Dtp, IPV, HBV, HIB, and MPRV. The HIB vaccination was not required for adults or for patients older than 17 years. In Italy, in 2022, a total of 191 cases were notified (rate 0.3/100,000 inhabitants) according to an ECDC report [19], with a high vaccination coverage (between 93% and 96%).

This study also showed very low coverages prior to a splenectomy, but it can be justified by frequent emergency admission, which cannot allow for planning a vaccination program before surgery. However, there is no consensus about the best timing for vaccination prior to a splenectomy [20]. It is clear that a vaccination program should be started as soon as possible prior to surgery. So, a vaccination protocol should be implemented by surgeons in order to direct patients who are candidates for a splenectomy to a vaccination center prior to surgery. Reaching target persons during their hospitalization is an opportunity to offer vaccination, especially among frail patients such as those who are splenectomized. An active-offer model of vaccination directly in a hospital ward can represent additional value in reaching people with recent episodes of illness who urgently need to be included in the vaccination schedule. So, booking an appointment directly at the time of discharge could help in improving vaccination among the splenectomized [21].

Also, general practitioners and other healthcare professionals should be involved in the vaccination program of splenectomized patients in order to improve vaccination coverages [22]. However, healthcare workers (HCWs) from other disciplines should improve their knowledge on vaccinology. Frequently, HCWs show low vaccination coverages due to low knowledge [23]. So, healthcare authorities must commit and make an effort to improve this knowledge gap. Improved medical data-sharing strategies between healthcare professionals would improve health outcomes of asplenic patients.

In order to improve coverages, it could be useful to also develop focused vaccination strategies for particular risk groups, such as oncological and hematological patients who frequently undergo splenectomy. For this reason, shared organization with a clinician could help in achieving better protection for fragile patients. Also, an information campaign leading to HCWs and splenectomized patients’ empowerment could help in improving vaccination coverages.

This study presents several strengths: firstly, this is the first study from the Abruzzo region reporting data on vaccination coverages among splenectomized patients. Secondly, it covered a large study period (from 2015 to 2023), using a standardized and deeply used source such as the HDR and RVR. Also, poor studies evaluated data on most recent years. However, some limitations should be considered. The HDR was lacking in some clinical information, such as drug therapy or comorbidities, which can be useful for regression analysis. In addition, some diagnosis codes could be miscoded or underreported. Information on out-of-hospital mortality or transfer to another Italian region cannot be caught by the HDR and RVR, so the vaccination coverage could be not precise. The possibility of enlarging the coverages to other regions could help in improving study results. In addition, patients with clinical asplenia cannot be evaluated because these data are not available on the RVR, and they are frequently underreported in the HDR. So, the main implication of these points is the underestimation of patients with hyposplenism and vaccination coverages.

## 5. Conclusions

In conclusion, coverages among splenectomized patients in the Pescara province are not satisfying but are over the values reported in other studies performed in other Italian regions and other countries. Unvaccinated patients are at high risk of developing severe infectious diseases. It is imperative to implement educational measures for patients and physicians to increase vaccination coverages. For this aim, it is mandatory to integrate the hospital setting with community care in order to improve the protection of this high-risk population. A shared organization that considers the clinician and general practitioner could help in improving patient protection against infectious diseases and mortality.

## Figures and Tables

**Table 1:** Patients’ characteristics.

N = 97	*n* (%)
**Gender**	
Male	60 (62.5)
Female	37 (37.5)
**Age class**	
<18	2 (2.1)
18–65	53 (54.6)
>65	42 (43.3)
**Admission**	
Emergency	61 (63.5)
Planned	36 (36.5)
**Diseases**	
Solid tumor	45 (46.4)
Trauma	38 (39.2)
Others	14 (14.4)
**Discharge**	
Death	16 (15.6)
At home	81 (84.4)

**Table 2:** Vaccination coverages before and after splenectomy.

N = 97		Men ACWY (2 Doses) *n* (%; 95%CI)	Men B (2 Doses) *n* (%; 95%CI)	PCV (13valent + 23valent or 20 Alone) *n* (%; 95%CI)	Hib (1 Dose) *n* (%; 95%CI)	COVID-19 (3+ Doses) *n* (%; 95%CI)
**Before splenectomy**	Fully vaccinated	21 (21.6%; 13.4–29.8)	5 (5.2%; 0.8–9.6)	5 (5.2%; 0.8–9.6)	13 (13.4%; 6.6–20.2)	NA
	Partially vaccinated	3	1	5	-	-
**After splenectomy**	Fully vaccinated	57 (58.8%; 49.0-68.6)	57 (58.8%; 49.0-68.6)	75 (77.3%; 68.4–85.6)	52 (53.6%; 43.7–63.5)	89 (91.8%; 86.3–97.3)
	Partially vaccinated	45 (46.4%; 36.5–56.3).1)	45 (46.4%; 36.5–56.3).1)	32 (33.0%; 23.6–42.4)	-	7 (7.2%; 2.1–12.3)
***p*-value ^**		<0.001	<0.001	<0.001	<0.001	

^ *p*-value refers to the comparison among fully vaccinated patients performed with a Chi-squared test. Percentage and 95%CI were reported only for classes with more than 10 cases according to the reviewers’ recommendations.

**Table 3:** Vaccination coverages by age classes.

N = 97	Men ACWY (2 Doses) *n* (%; 95%CI)	Men B (2 Doses) *n* (%; 95%CI)	PCV (13valent + 23valent or 20 Alone) *n* (%; 95%CI)	Hib (1 Dose) *n* (%; 95%CI)	COVID-19 (3+ Doses) *n* (%; 95%CI)
**<18 (N = 2)**					
**Fully vaccinated**	2	2	2 *	2 *	1 (50%; 40.1–59.9)
**18–65 (N = 53)**					
**Fully vaccinated**	34 (64.2%; 54.7–73.7)	34 (64.2%; 54.7–73.7)	43 (81.1%; 73.3–88.9)	17 (32.1%; 22.8–41.4)	46 (86.8%; 80.1–93.5)
**Partially vaccinated**	20 (37.7%; 28.1–47.3)	5 (9.4%; 3.6–15.2)	13 (24.5%; 15.9–33.1)	-	7 (13.2%; 6.5–19.9)
**>65 (N = 42)**					
**Fully vaccinated**	21 (39.6%; 26.4–52.8)	21 (39.6%; 26.4–52.8)	30 (71.4%; 62.4–80.4)	33 (78.6%; 70.4–86.7)	42 (100%)
**Partially vaccinated**	25 (47.2%; 34.0–60.4)	25 (47.2%; 34.0–60.4)	13(30.9%; 21.7–40.1)	-	-
***p*-value ^**	<0.001	<0.001	<0.001	<0.001	<0.001

* Both patients received three doses of PCV and Hib as requested by the pediatric schedule during the first year of age. ^ *p*-value refers to the comparison among fully vaccinated patients performed with a Fisher’s Exact test. Percentage and 95%CI were reported only for classes with more than 10 cases according to the reviewers’ recommendations.

**Table 4:** Factors associated with full vaccination.

	*n*	MenACWY aOR (95%CI)	*n*	MenB aOR (95%CI)	*n*	PCV aOR (95%CI)	*n*	Hib aOR (95%CI)	*n*	COVID-19 aOR (95%CI)
Male gender	32	1.08 (0.76–1.45)	32	1.07 (0.76–1.47)	40	1.13 (0.81–1.59)	38	1.10 (0.80–1.57)	59	1.01 (0.69–1.86)
Age	53.2	1.02 (0.87–1.47)	53.2	1.02 (0.87–1.48)	54.6	1.03 (0.91–1.38)	53.6	1.04 (0.90–1.41)	53.2	1.10 (1.01–1.29) *
Trauma	28	1.05 (0.90–1.22)	28	1.04 (0.89–1.24)	26	1.00 (0.77–1.60)	38	1.05 (1.00–1.13) *	37	0.99 (0.76–1.65)

Abbreviations: aOR = adjusted odds ratio; 95%CI = 95% confidence interval. * *p*-value < 0.05.

## Data Availability

Data were not available due to privacy restrictions.

## Supplementary Materials

The following supporting information can be downloaded at: https://www.mdpi.com/article/10.3390/vaccines13020138/s1, Table S1: Factors associated to vaccination status.
