# Supplementary material for: Vaccination Coverages Among Splenectomized Patients: A Retrospective Study from an Italian Southern Province
Source: Vaccines (Basel). 2025 Jan 28;13(2):138. doi: 10.3390/vaccines13020138 (PMC11860258; doi:10.3390/vaccines13020138)
Supplement: Supplementary file 1 [file vaccines-13-00138-s001.zip › vaccines-3405011-supplementary.pdf]

Table S1. Factors associated to vaccination status

		MenACWY RRR(95%CI)	MenB RRR(95%CI)	PCV RRR(95%CI)	Covid19 RRR(95%CI)
Partially vaccinated	Male gender	1.50 (0.16-13.81)	1.79 (0.68-1.99)	1.11 (0.08-8.11)	0.96(0.09-4.84)
	Age	0.99(0.91-1.07)	1.09(0.99-1.19)	0.97(0.89-1.06)	0.99(0.84-1.08)
	Trauma	1.01(0.67-2.95)	1.02(0.59-3.02)	0.99(0.60-2.98)	1.04(0.72-2.88)
Fully vaccinated	Male gender	1.74(0.20-15.33)	1.79 (0.21-17.23)	1.27 (0.18-8.11)	0.89(0.09-4.84)
	Age	0.99(0.92-1.07)	0.99(0.92-1.07)	0.96(0.88-13.72)	1.00(0.84-1.04)
	Trauma	1.01(0.88-2.55)	1.00(0.82-2.66)	1.00(0.90-2.58)	1.03(0.79-2.69)

Abbreviations: RRR= relative risk ratio; 95%CI= 95% confidence interval
